# The Research Trend of Big Data in Education and the Impact of Teacher Psychology on Educational Development During COVID-19: A Systematic Review and Future Perspective

**DOI:** 10.3389/fpsyg.2021.753388

**Published:** 2021-10-27

**Authors:** Jia Li, Yuhong Jiang

**Affiliations:** Foreign Languages College, Shanghai Normal University, Shanghai, China

**Keywords:** data science applications, bibliometric study, research trend, teacher identity, geographical diversity, educational development

## Abstract

The COVID-19 outbreak, along with post-pandemic impact has prompted *Internet Plus* education to re-examine numerous facets of technology-oriented academic research, particularly Educational Big Data (EBD). However, the unexpected transition from face-to-face offline education to online lessons has urged teachers to introduce educational technology into teaching practice, which has had an overwhelming impact on teachers' professional and personal lives. The aim of this present work is to fathom which research foci construct EBD in a comprehensive manner and how positive psychological indicators function in the technostress suffered by less agentic teachers. To this end, CiteSpace 5.7 and VOSviewer were applied to examine a longitudinal study of the literature from Web of Science Core Collection with the objective of uncovering the explicit patterns and knowledge structures in scientific network knowledge maps. Thousand seven hundred and eight articles concerned with educational data that met the criteria were extracted and analyzed. Research spanning 15 years was conducted to reveal that the knowledge base has accumulated dramatically after many governments' initiatives since 2012 with an accelerating annual growth and decreasing geographic imbalance. The review also identified some influential authors and journals whose effects will continue to have future implications. The authors identified several topical foci such as data mining, student performance, learning environment and psychology, learning analytics, and application. More specifically, the authors identified the scientific shift from data mining application to data privacy and educational psychology, from general scan to specific investigation. Among the conclusions, the results highlighted the important integration of educational psychology and technology during critical periods of educational development.

## Introduction

Educational Big Data (EBD) is currently faced with an unprecedented recognition of existing educational psychology, with technological platforms playing an increasingly vital role in the adaptation of current approaches toward technology-based programs. EBD has emerged as a vital area of study for both educators and researchers, reflecting the magnitude and impact of data-related problems to be solved in educational practices, particularly with the application of innovational technologies. Nowadays, recreational desires, commercial insights, research needs, and government initiatives necessarily accelerate the utilization of technological devices, producing a great amount of data on an unprecedented scale. For better and for worse, the accumulation and circulation of massive data on each form have become an integral part within the development of contemporary social community. It is a topic that merits the close focuses of all walks of life, especially those in academic research. To analyze and further dig out the underlying function of big data for both public and private benefit, researchers from different domains have tried to unpack and define big data in increasingly powerful ways (Mikalef et al., 2018).

Back to 2012, the need for research on the previous large volume of human experience to improve the working efficacy and well-being for offspring stood out. This demand produced the idea of BIG DATA as massive quantities of information produced by humanity, surroundings, and their interrelations (Boyd and Crawford, 2012). Big data has several characteristics known as “5V”: *Volume, Velocity, Variety, Veracity, and Value* (Demchenko et al., 2013). *Volume*, one of the characteristics of big data, indicates the amount of data is huge and unpredictable along collecting, restoring, and calculating. *Velocity* introduces one nature of big data. It calls for fast processing to online or real-time data analyses, which also requires the unique data mining technology different from traditional ones. *Variety* is the basic concept in big data, referencing a variety of data sources (including semi-structured and unstructured data) and the data types and formats breaking through the traditional limited category of structured data, either structured or unstructured. *Veracity* refers to the quality of data. When the source becomes more complicated and diverse, the truth and reliability need to be further analyzed. Finally, *value* mentions the laborious input that would bring the high value in return. Similarly, Saggi and Jain (2018) added two more characteristics, namely *Valence* and *Variability*. However, as large-volume, intricate, growing data assets from a variety of sources, analyzing big data in traditional manner is a challenging but fruitful work (Wu et al., 2013; Osman, 2019).

Currently, several scholars, such as Frizzo-Barker et al. (2016) have become more involved and thrilled about the feasibility of big data. Actually, the appeal of big data has never been lost in many different realms such as economics (Varian, 2014), business (McAfee et al., 2012), ecology (Hampton et al., 2013), geography physical (Li et al., 2016), medical care (Liao et al., 2018), and health care sciences services (Bates et al., 2014). Moreover, in these research fields, the research method of systematic reviews has already been adopted to provide broader assessment (Connolly et al., 2012; Perez et al., 2013; Rose et al., 2018).

Additionally, despite the rapid application of science mapping in the domain of information science, social science, and medical research, the utilization of comprehensive visualization networks to better understand the evolution of Educational Big Data is quite novel (Eynon, 2013). Despite the exponentially increasing growth and interest among participants and scholars, there is not enough research on big data in education, especially with the application of systematic bibliometric analysis. Our primary objective of the visual analysis is to apply data mining technology to excavate high-quality and effective information from data and use informative pictures to clearly display research achievements in the field of education based on *Authors, Countries, Journals, Institutions, Key Words*, and *Research Topics*. This allows us to faster capture changes across multiple data sets without the need to acquire sophisticated computer skills or master clustering techniques (Van Eck and Waltman, 2017). Finally, it would provide insights for future studies and highlight the potential directions for the big data in education. Therefore, a macroscopic overview needs to be available on the main characteristics based on the bibliometric review.

## Literature Review

Initiated 15 years ago, educational big data has drawn many educational scholars' attention and gained abundant academic achievements. The educational realm has never lost its crucial role with the advent of big data. Much data in the field of education has increased significantly since the release of the Internet and researchers can explore some groups of subjects without necessarily depending on complicated measuring methods. Earlier, gStudy and learning kits were utilized as a medium through which learners construct knowledge and produce more informative data about knowledge construction in psychology (Winne, 2006). In the age of big data, which provides educational scholars with comprehensive ways to reconceptualize research questions and analyze educational data (Daniel, 2015), technological tools are applied to collect useful data within short time and relatively low cost (Mayer-Schönberger, 2016). Software technologies in education contribute greatly to big data and improve learning for the better and promote school reform based on the three axioms in educational psychology, respectively, “Learners Construct Knowledge (Cognitive Operations), Learners Are Agents (The capacity to exercise choices with respect to preferences), and Data Include Randomness” (Winne, 2006). However, in the technology-based teaching environment, it is agentive teachers who play an important role in applying educational technology in their teaching practices and they determine which methods are used to construct the classroom pattern and how to execute teaching plan in more effective ways. The need to focus on teachers' psychology in educational development emerges during investigation.

Online course, instruction, and guidelines produce a considerable amount of educational data, which provides teachers with the access to student's performance and learning patterns (Oi et al., 2017). Those data could help teachers to further analyze students' learning route and teaching pedagogy (Holland, 2019). Visualization techniques were suggested to capture and identify available and fruitful patterns in educational data (Greer and Mark, 2016). For instance, a newly appointed math teacher can utilize visualization tools and test data to know in which branch a student performs better, statistics or geometry. Therefore, visualization outputs have an ability to help teachers with limited disciplinary knowledge to interpret and unscramble student data (Ong, 2015). Currently, it is also necessary and important for educational scholars to realize what big data really means for education.

## Research Design

### Research Questions

Based on the bibliometric research of big data in education psychology, the main research questions can be uncovered based on the statistics from databases. Moreover, in the light of this systematic investigation, some insights into educational reforms in school and implications for teachers and teaching could be unveiled, and also the need to fathom how educational technology affects teachers' actions psychologically in teaching stands out.

- What is the growth trajectory and geographic distribution of literature in the domain of EBD from 2006 to 2021?
- What main research foci and trends have gained the greatest attention from the clustering analysis?
- What implications and insights for teachers and teaching could be acquired from the literature review of EBD?

### Materials and Methods

**Figure 1:**
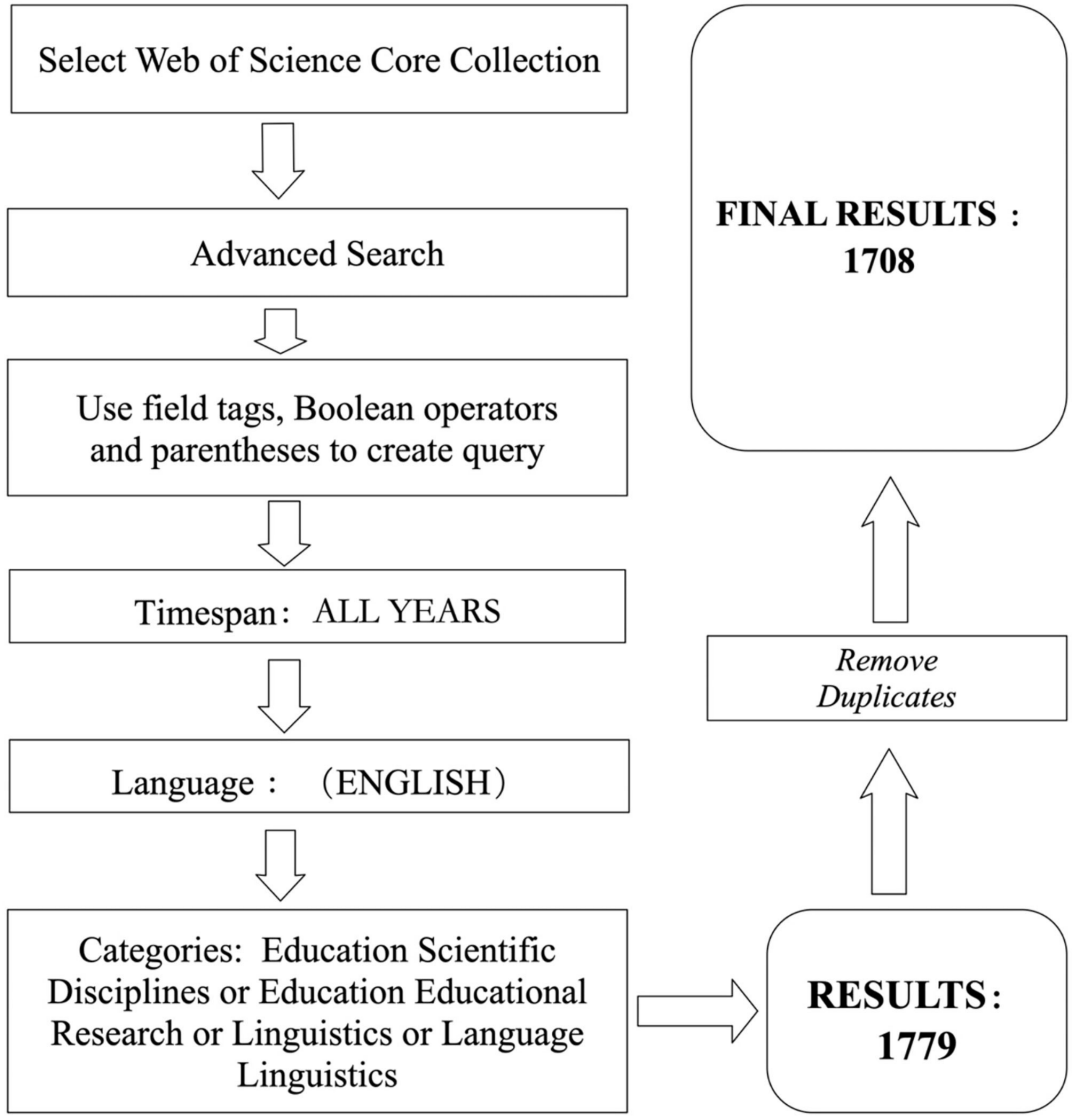
Stages of data collection.

The data in this paper was obtained from Web of Science (WoS) on February 5, 2021. WoS is naturally regarded as the world's largest comprehensive academic information research database covering more than 8,700 core academic journals. In order to retrieve more high-qualified articles, the authors selected core collection as research objectives. Details of collection of data are shown in Figure 1.

In this study, related keywords were listed and used to complicate the Boolean logic models: TS = (“Big Data” Or “Learning Analytics” Or “Data Analytics” Or “Data Mining” Or “Big Data Era” Or “Data Models” Or “Data Management”) And TS = (“Education” Or “Language” Or “Learning” Or “Educational Psychology” Or “Educational Application”), and according to returned results, the first publication in education appeared in 2006. After running the process, 1,779 items met the selection criteria. Up to the date of analysis, the results show that article (1,643, 92.4%) enjoys the most frequent document type, the second is review (65, 3.7%), and at the third position is editorial material (62, 3.5%). Other types include book review (6, 0.3%), correction (2, 0.1%), and software review (1, 0.05%). After discarding duplicate data, the number of total unique records is 1,708.

As the most frequently used tool in bibliometrics, science mapping presents the current status of research and possible developmental directions. In this paper, bibliometric software VOSviewer and CiteSpace (Chen, 2006) are utilized for data analysis. Bibliometric software CiteSpace provides effective methodology in systematic scientometric review (Chen and Song, 2019), which specializes in analyzing keywords timeline picture for possible research direction. During the process of keywords visualization, time span was set to “2006 to 2021,” time slice was one year, node type could be confined to analysis-preferred themes (*author, organization, reference, et al*.) and other parameters were set to default values. The results would be presented in the next discussion part. Apart from timezone analysis, VOSviewer is another bibliometric mapping software used for co-occurrence and co-citation analysis (Van Eck and Waltman, 2010). Prior to data processing, filtered data would be imported into network dataset for visualizing and exploring maps. To illustrate and satisfy different research objectives, network, overlay and density visualization are available for data representation. The keywords could be distributed as nodes in display in the three maps (*open* button on the file tab in the action panel). In the function panel, different outcomes could be scrutinized based on the different subjects, which are shown in the discussion section.

CiteSpace as well as VOSviewer utilizes nodes to represent keywords and lines as co-occurrence relationship, which could be visualized in the form of graph structures. However, these two pieces of bibliometric software enjoy different priority in data processing due to the nuance of theoretical algorithm. It is acknowledged that CiteSpace has the power to better display the development trend in the specific research realm and forms the frontier of research evolution. VOSviewer, on the other hand, concentrates on the display of main information retrieved from database. Hence, in this research review, timeline and keywords burst would be analyzed using CiteSpace, and co-citation display of different sections would be managed by VOSviewer.

## Research Results

In this part, the authors present the results of publications in big data in education in a comprehensive bibliometric way. A state of the art in education is presented in A state of the art in EBD study. Further, keywords analysis of research foci, co-authorship and co-citation analysis are shown respectively.

### A State of the Art in EBD Study

In this section, the authors analyze the current status of study from different aspects, including annual trends of publications, the distribution of institution and journals, and citation.

#### The Annual Trends of Publications of EBD

**Figure 2:**
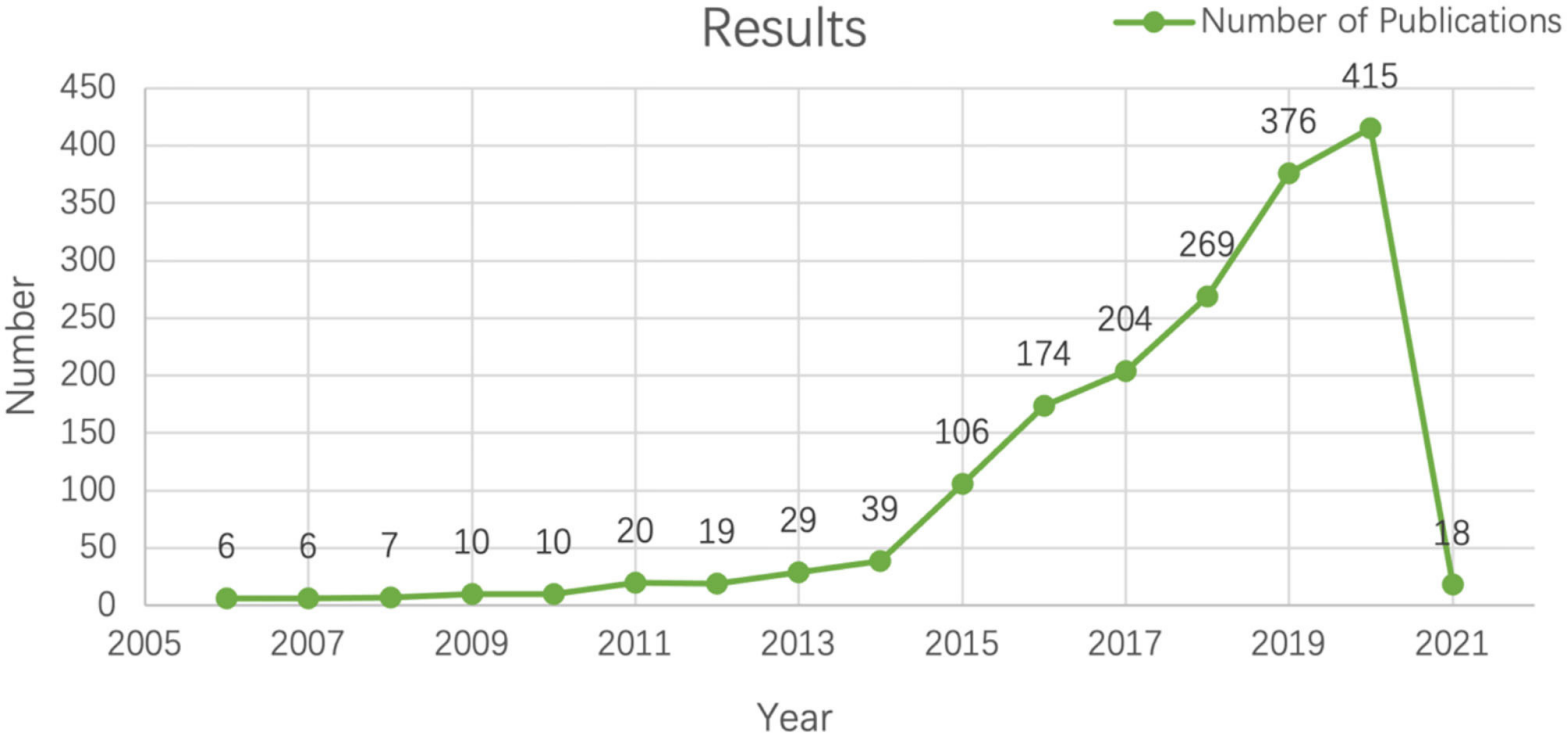
The annual trends of publications of *big data in education* based on WoS core data base.

After the application of big data in education, 1,708 papers were published on the Web of Science core collection from its inception in 2006 to February 5, 2021. The annual trend of these publications is demonstrated in Figure 2. The graph shows that there are three stages from 2006 to 2021. In the first stage, 2006 to 2010 (the figures under 10), the study of big data in education is still in the initial stage. From 2011 (20) to 2014 (39), in spite of some subtle declines, the number of publications slightly increases compared to 2006 (6). After 2014 (39), the figure shows dramatic growth. Up to 2020, the figure rises to 415. This significant growth trend signals that big data in education has drawn more and more scholars' attention and probably continues to increase in the next two decades.

#### The Distribution of Influential Institution on EBD

**Figure 3:**
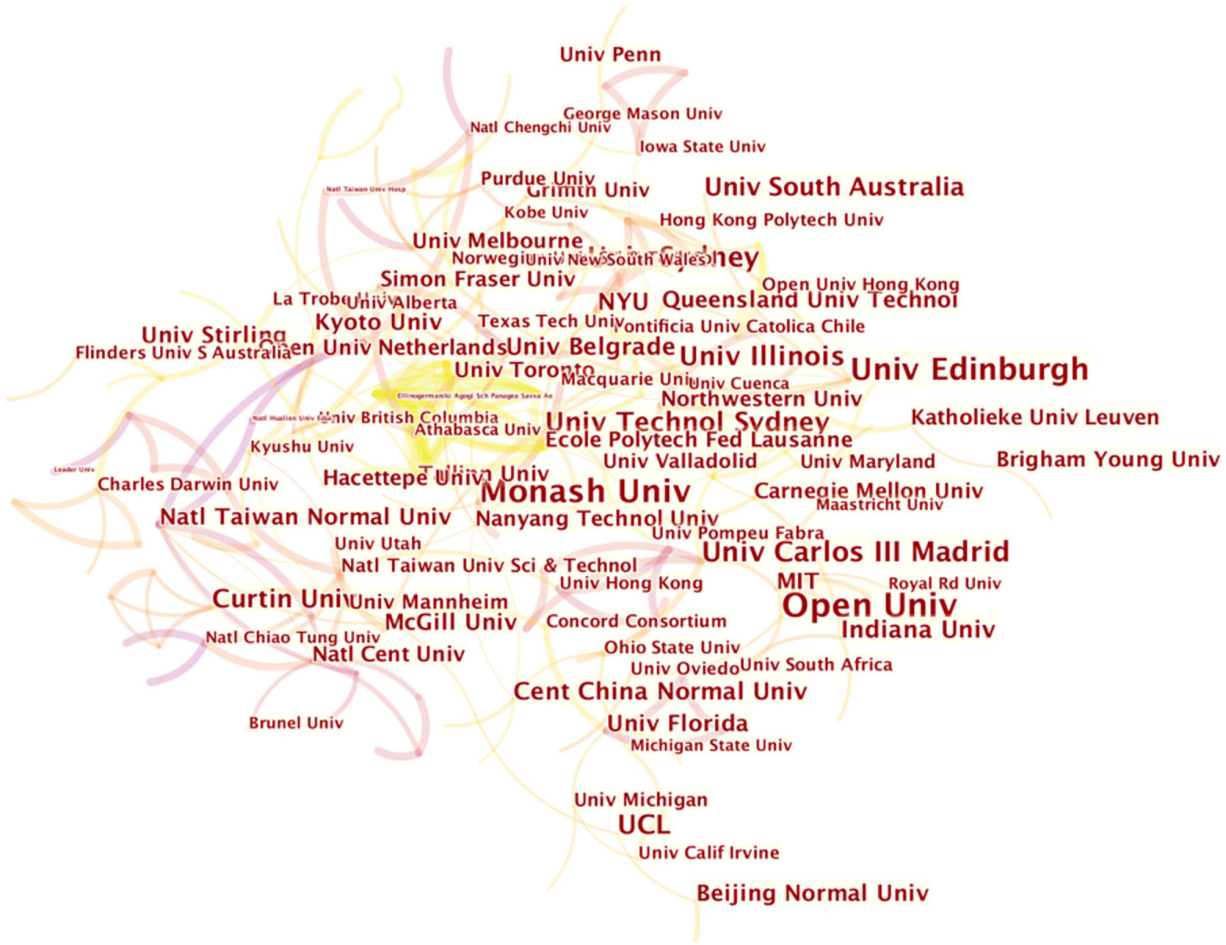
Institution co-occurrence network of big data in education.

We utilize CiteSpace to get the knowledge map of institution co-occurrence network (as shown in Figure 3). The results show that the whole graph network is distributed densely. Moreover, there are many connections between each node, which indicates that scholars in the study of big data in education cooperate closely, and most of the research is cooperative research.

Further, to further analyze the prominent institutes in the domain of education, select *network summary table* in CiteSpace and get the top dominant results (See Table 1). It shows that Open University enjoys the priority of greatest numbers of publications, which has 41 papers in the field of educational big data. Not like traditional face-to-face models, OU attaches great importance to e-learning for the purpose of flexibility. Lara et al. (2014) from OU applied educational data mining and other major strengths to meet the challenge of the spatial and temporal gap between students and teachers. The Monash University (Australia) is at the second position and has totally published 36 papers followed by the Edinburgh University (33), the Sydney University (22) and the Carlos III Madrid University (22) respectively. From the listed 10 organizations below, the clear and plain fact shows the number of each individual's publications is <50 papers, which indicates that in the domain of education, big data is still a niche topic.

**Table 1:** Different analytical tools in data processing.

**Thematic Distribution**	**Analytical Tool**
Distribution of influential institutions	CiteSpace
Keywords co-occurrence analysis	VOSviewer
Keywords timeline view	CiteSpace
Keyword citation bursts	CiteSpace
Co-authorship visualization analysis (including geography, organizations, reference and journal)	VOSviewer

*TLS, total link strength; APY, average publication year; IF, impact factors*.

#### The Analysis of Citation and H-Index

**Figure 4:**
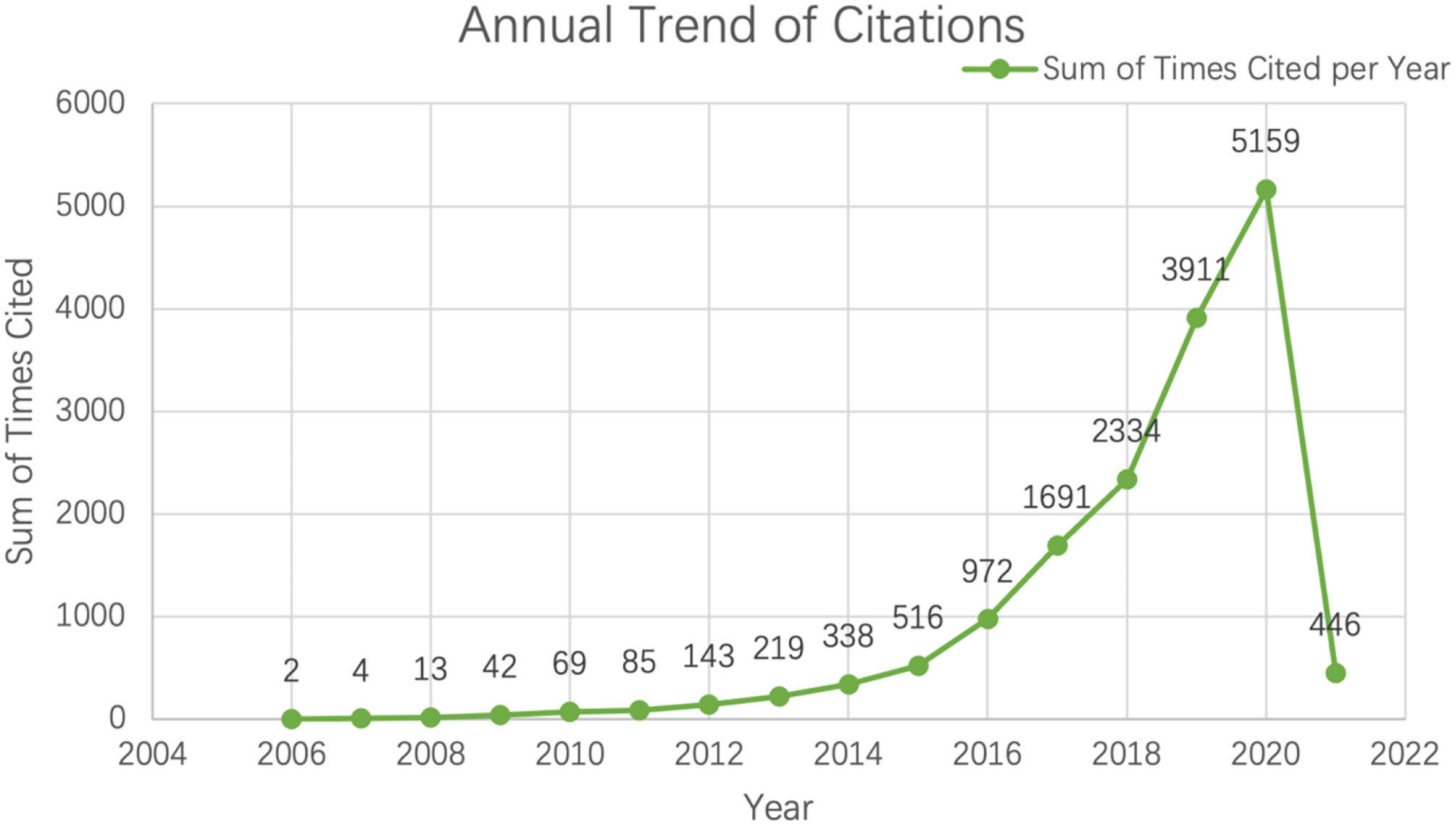
Sum of times cited each year on web of science.

In order to achieve higher impact on the scientific community, scholars often want to publish their findings in some certain high-impact journals (Bhandari et al., 2007). The number of citations becomes the main indicator to access the quality of a paper (Tahamtan et al., 2016). Web of Science has its own analyzing tools to create citation report, which could reflect citations to source items. From core collection between 2006 to 2021, the total number of citations is 15,944 (see Figure 4) and the number of “without self-citations” is 12,277.

Hirsch (2005) originally coined *H-index* to access the one person's academic achievement and further indicated that if a researcher's total papers have at least *h* citations each and the other outputs have <*h* citations each, then he or she has factor *h*. From the citation report from Web of Science, what can be concluded is that the *h*-index of research results is 51 and average citations per item is 8.93. According to the findings of citation and *h*-index, although compared with medica and information science, the integration of big data with education is a relatively new topic, great attention has been caught in this field.

### Keywords Analysis of EBD

This section provides the scientific landscapes of keywords in educational big data. The keywords co-occurrence network map, the density visualization map and timeline map will be exhibited by using VOSviewer bibliometric software. Further, with the help of CiteSpace, the table of citation bursts will be displayed respectively.

#### Keywords Co-occurrence Network

How academic knowledge is stored and evolved over time is an intriguing question. New ideas and findings cannot be kept separate from existing principles and concepts (Palvia et al., 2002; Oh et al., 2005). The structure of knowledge and its variations are interrelated within social community, which makes network perspective available in study. For the sake of convenience and effectiveness, some keywords serve as an indicator of the significance of research topics (Choi et al., 2011). Therefore, the analysis of keywords occurrence network could report research hotspots and future trends of some certain realms to some extent.

**Figure 5:**
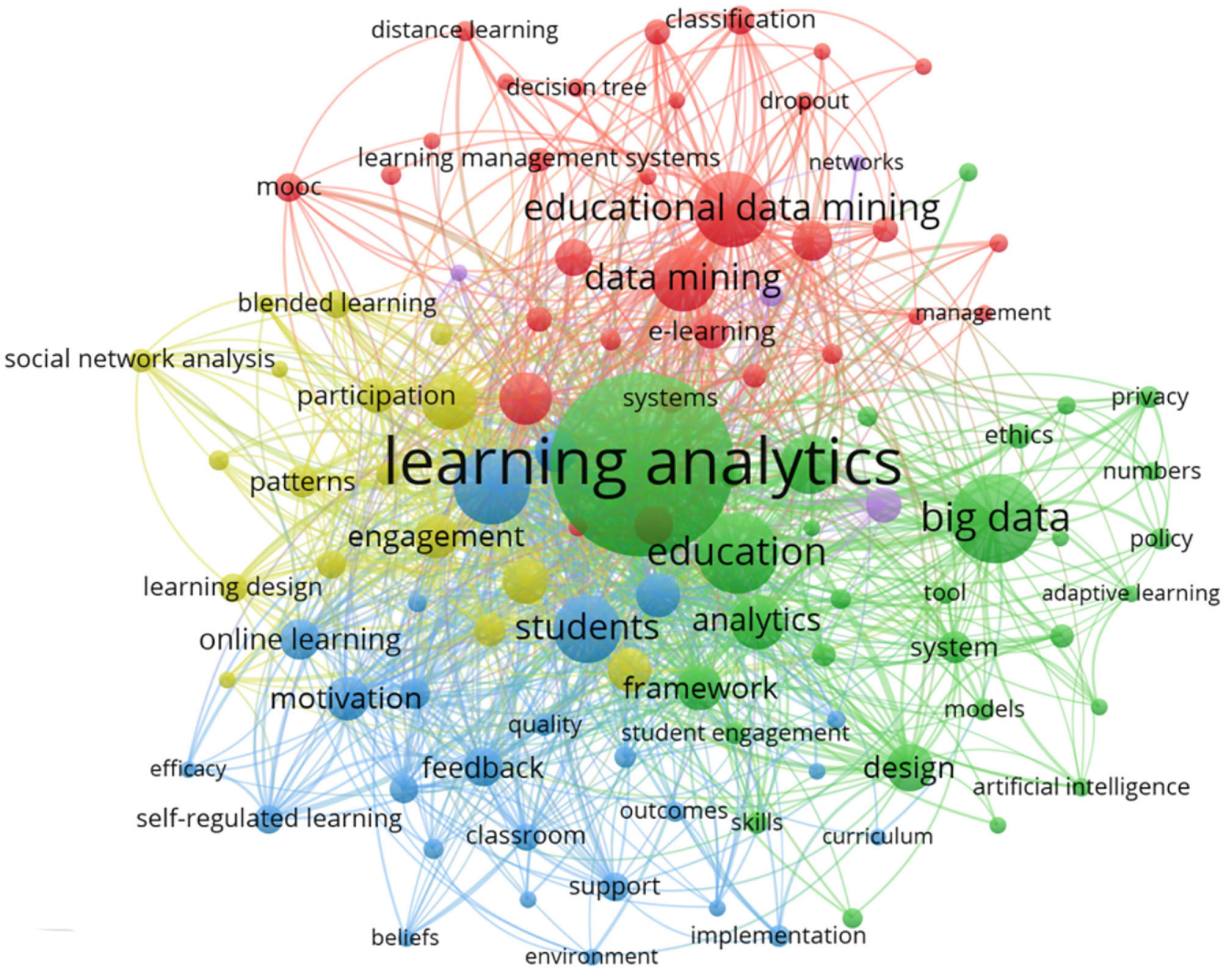
Keywords network visualization of big data in education.

After importing network data into VOSviewer software, 5,229 keywords were obtained. Further, the threshold of minimum occurrences was set as 15 and the keywords with the greatest total link strength were selected to create a network visualization map (see Figure 5). According to the manual of VOSviewer 1.6.16, the size of the nodes stands for the occurrences and weights of the keywords. If one item has the biggest circle, the largest weights it has. The distance between two words represents their relations in the intensity distribution. The shorter distance two words has, the stronger their relatedness. Moreover, nodes with the same color represent that they are in the same cluster (Van Eck and Waltman, 2010). Hence, from the distribution of keywords in the map, it is clear to see that the biggest node is “learning analytics” which appears 630 times. “Big data” (203), “Education” (177) and “Educational Data Mining” (160) disclose their occurrence in the study respectively.

Further, the whole network occurrence map could be divided into five clusters. Each one represented a distinct branch of big data in education. To be specific, in the red cluster (cluster 1, 31 items), keywords such as *Educational Data Mining, Data Mining, Model, Machine Learning, MOOC* (Massive Open Online Course), *E-Learning, Learning Management Systems*, etc., focused on the research of data mining and application in education. The green cluster (cluster 2, 30 items) included the keywords such as *Learning Analytics, Big Data, Education, Analytics, Design, Tools, System, Ethics, University*, etc., which were concerned with learning analytics. Another blue cluster (cluster 3) consisted of 26 keywords, including, *Efficacy, Motivation, Online Learning, Belief, Support, Self-Regulated Learning, Student Engagement, Environment*, etc. The blue cluster unveiled the importance and potential of psychological factors in the language teaching and more specifically teachers' development. Next, in the yellow cluster (cluster 4, 17 items), keywords like *Engagement, Patter, Social Network Analysis, Participant, Blending Learning, Learning Design*, etc., showed the common feature as learning environment and patter. There were four items in the last purple cluster (cluster 5), *Facebook, Networks, Science*, and *Social Media*, which implied the source of educational big data.

To be more specific, the information of top 10 keywords with their occurrence, links and total link strength are displayed in Table 2. The link strength and total link strength are another two indexes to quantify the relatedness of keywords (Pinto et al., 2014). The first index appertains to frequency of co-occurrence and the total link strength refers to the sum of the link strength of the keywords. Based on the Table 2, apart from big data and education, keywords like learning analytics, data mining, students, online and performance enjoy the privilege of co-occurrence.

**Table 2:** The top institutes with big data in education publication.

**Organization**	**Freq**	**Centrality**	**Year**
Open Univ	41	0.03	2007
Monash Univ	36	0.17	2011
Univ Edinburgh	33	0.11	2015
Univ Sydney	22	0.01	2014
Univ Carlos III Madrid	22	0.04	2010

**Figure 6:**
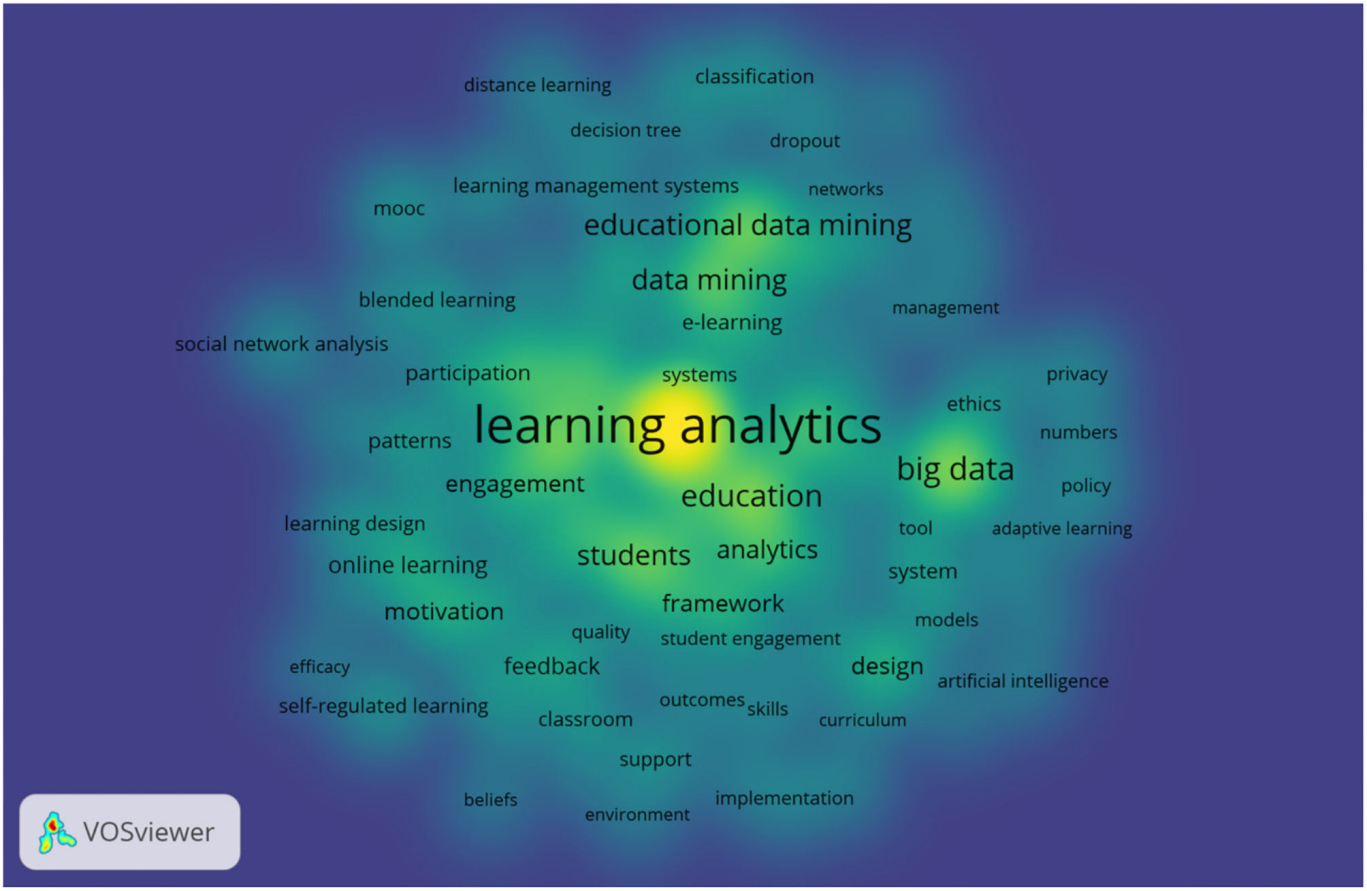
Keywords density visualization of big data in education.

VOSviewer can also export the map of density visualization (see Figure 6). According to Van Eck and Waltman (2011), keywords in the map have the similar way as in the network visualization. Each item owns its self-color to identify the density of keywords at that point. By default, blue, green and yellow are the three main colors to show the distribution of density. The larger the number of the items in the neighborhood of the node, the more frequently the keywords appear and the closer the color is to yellow and vice versa. From the output of density visualization map, learning analytics, big data, education, educational data mining and students have the most crucial impact in the field of educational big data.

#### Keywords Timeline View of EBD

**Figure 7:**
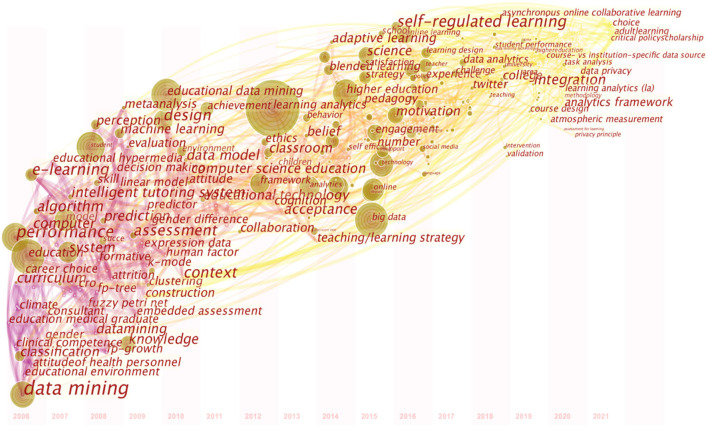
Keywords Timeline view based on CiteSpace.

The software CiteSpace has the keyword-analyzing capacity for unveiling the cutting-edge research by presenting the certain research contents and distribution of some certain topics over time (Chen, 2006). Based on the keywords co-occurrence map, the authors selected the “time zone” option in the control panel and got the output (Figure 7).

From the output of CiteSpace, Table 3 lists the main keywords appeared during the different periods.

**Table 3:** The details of top 10 occurrence keywords.

**Rank**	**Label**	**Occurrences**	**Cluster Number**	**Links**	**TLS**
1	Learning analytics	630	2	106	1,816
2	Big data	203	2	87	419
3	Education	177	2	102	555
4	Educational data mining	160	1	94	523
5	Performance	158	3	98	744
6	Students	131	3	97	530
7	Data mining	124	1	87	259
8	Higher education	110	2	90	371
9	Online	98	4	93	459
10	Analytics	93	2	93	365

Obviously, from the distribution of keyword nodes, it can be spotted that there are three distinct time zones from starting year 2006 to 2021. For the sake of convenience, the authors separate the whole timeline into three periods, namely “Incubation Period,” “Boom Period” and “New Stage of Incubation Period” respectively.

In the period of incubation (2006–2011), the study on educational big data is little, and the products are relatively immature. The keywords in that period focused on “data mining,” “algorithm,” “computer” and “educational environment.” Realizing the potential of big data, scholars in the realm of education have made great efforts to exploit the application of technology to utilize and analyze the massive valuable data in powerful ways. They adapted themselves to the era of big data. The research in this period paved the way for the further investigation of big data in education. For instance, in 2008, Romero, Ventura and Garcia conducted a survey of application of the data mining tool in learning management systems and introduced to all potential administrators, which opened the door of educational data mining.

After 2011, the study enjoyed a period of prosperity (2012–2016). More and more eminent scholars and experts treated data from educational context as a valuable way to trace students' performance. Big data has become a research focus in the field of education. The publications begin to accumulate and the keywords like “learning analytics,” “classroom,” “blended learning” and “self-regulated learning” direct the way to integration of big data and education. In the paper, named *Translating Learning into Numbers: A Generic Framework for Learning Analytics*, Greller and Drachsler (2012) investigated the main dimensions of learning analytics to design a practical framework in support of educational implementation and teaching efficiency.

After 2016, the investigation entered the new round of incubation. In this period, the keywords like “data analytics,” “challenge,” “data privacy” and “course-design” account for the main proportions and the products of educational big data are gradually mature. Moreover, based on the keyword timeline map, it is clear to address that the sparks of new ideas are about to be kindled and there are two main directions: one is concerning data analytics providing the possibility of implementation, the other is about individual privacy issues. On March 19, 2021, the State Council Information Office (SCIO) held a press conference in Beijing on the fourth Digital China Summit. Yang, vice minister of the Cyberspace Administration of China, demonstrated the great importance attached to the publicity of data security and personal information protection, which underpinned the practicability of the nationwide enforcement of Network Security (Yang, 2021). The overall and coordinated efforts on various facets such as policy, law and supervision have been made to formulate national laws to provide legal protection for data security and personal privacy protection at the legal level.

From the trend of the research, the focus has shifted from technology-based investigation and practices to curriculum designing and subjects' self (psychological impact on teachers' professional development or “growing-up”). From the start, research has paved the way for the future analysis of educational practice in the school climate, then with the development and maturing of the educationally technological foundation, the main picture of the study has been transmitted to individual subjects, especially psychological factors. Recently, the combination of principles in cognitive psychology and education has shed light on the foggy investigation of psychology, such as students' attentiveness, teachers' agentic involvement as well as currency of mind wandering in educational settings (Szpunar et al., 2013).

#### Keyword Citation Bursts

**Figure 8:**
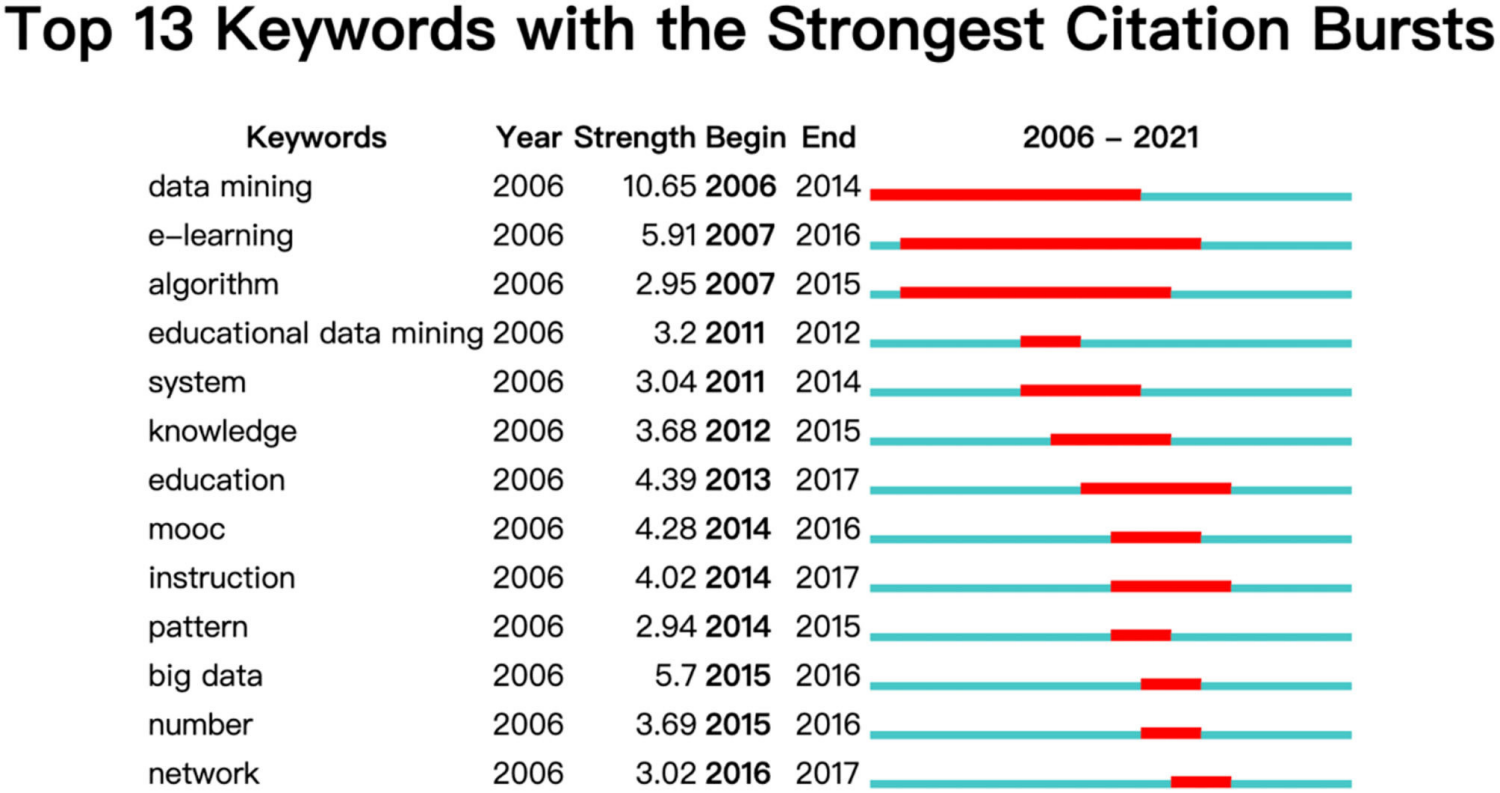
Results of strongest citation bursts in educational big data.

The trail of the scientific development can be traced from the keywords of the research works (Yang et al., 2020). Keywords with transition phenomenon have the way to unveil the implicit information of trends. The analysis of citation bursts can single out several keywords which has been paid special attention to by the related community within a certain period of time (Su and Lee, 2010; Chen et al., 2019; Su et al., 2019). Based on the powerful function of CiteSpace, this paper chooses the burst start time method to produce the top 13 keywords with strongest citation bursts (see Figure 8).

The red part in the figure shows the start year when the citation burst occurred. As can be seen from Figure 8, together with “e-learning” and “algorithm” which lasted for the longest time, the keyword “data mining” starting in 2006 was the first one to be proposed in the research of educational big data. The figure charts the dynamic transition from 2011 by inspecting the keywords order such as “system,” “education,” “patter,” “number” and “network.” Like Ozga's (Ozga, 2009) contribution to the prominent role of data in the use of benchmarking, performance criterion and monitoring within education context, Grek and Ozga's (Grek and Ozga, 2010) indigenous investigation of the European educational environment has pictured the inseparable relations between data and the education landscape, using data systems to track policy problems and develop policy solutions (Lingard et al., 2012). What is more, the Actor Network Approach is another policy-related factor concentrated on assemblages of human and non-human materials within any educational environment. Hence, from the message which the keywords order conveyed, the trend has flowed from individual performance evaluation to educational policy influence beyond the national scale due to the policy as numbers phenomenon and neoliberalism (Selwyn, 2015).

### Co-authorship Visualization Analysis

Academic research needs laborious engagement and investment, which means that it would be impossible for just one individual to accomplish a research project. Co-authorship has been used as an index for research collaboration by science policy academics and evaluators (Bond et al., 2019).

**Figure 9:**
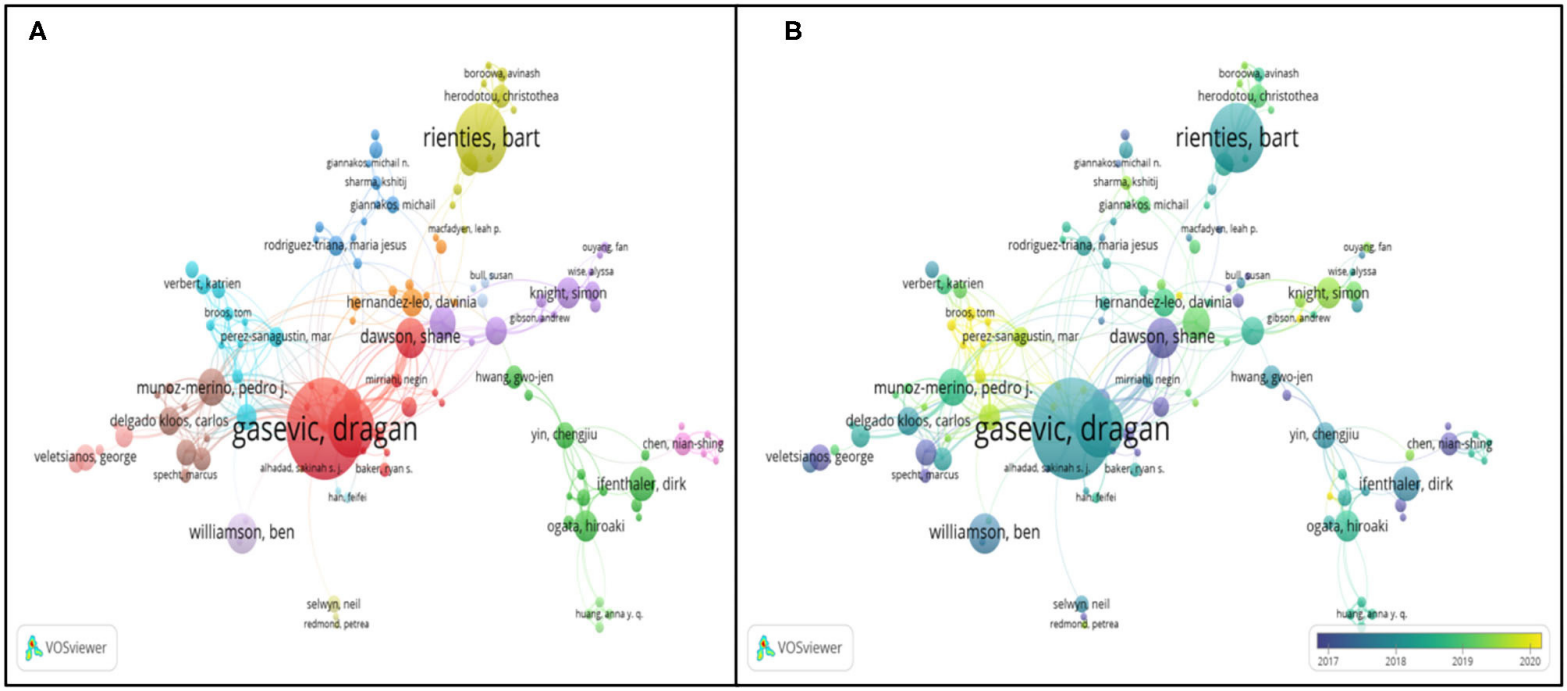
Authors cooperative network in the realm of big data in education. **(A)** Network visualization map based on link weights; **(B)** Overlay visualization map based on link weights.

In this section, VOSviewer was applied to investigate the collaborative pattern of author, country, and institution of big data in education. Inputting 1,708 items into software, the authors chose the unit of “authors” in the type of “co-authorship” and obtained the cooperative network of the authors in the field of educational big data (Figure 9). Of the 1,708 papers published between 2006 and 2021 by 4,380 authors, 633 authors (accounting for 14.45%), were credited on two publications, 246 contributors (5.62%) on three publications, and 126 contributors (2.88%) met the thresholds of four. In order to examine the prominent authors in the realm of educational big data, the authors set up the threshold of three. However, there were 124 items singled out without connections to other authors, leaving the rest of the 122 items to be analyzed in the network.

According to the manual of VOSviewer, lines between different contributors unveil the collaboration links, and several colors in the map represent the distinct clusters in the domain of educational big data. For instance, in Figure 9, the authors like “Yin, Cheng Jiu,” “Shimada, Atsushi,” “Ogata, Hiroaki,” “Chu, Hui Chun” and “Hwang, Gwo Jen” were grouped in the cluster 2 and highlighted in green.

In Figure 9B, the gradient colors disclosed an interesting trend of cooperation relatedness of contributors from single author to cooperative movement. The top productive authors “Gasevic, Dragan,” “Rienties, Bart,” “Dawson, Shane” and “Williamson, Ben” were in descending order, without latest contributions. Some authors like “Broos, Tom,” “Gentili Sheridan” recently have some new publications. For instance, in April 2020, Broos has realized the potential of learning analytics (LA), and proposed the coordination model to support the prosperous interaction between LA policymaking and implementation in Latin-America (Broos et al., 2020), which hopefully guided the futural LA initiatives. Later in June, Broos with other authors conducted the empirical investigation of learning analytics to improve academic support in Latin America (Guerra et al., 2020).

To make the Figure 9 more reliable in statistics, Table 4 lists the top document-productive authors. The average publication year shows that the top 10 contributors had publications after 2016. Combined with the annual trend of publications in Figure 2, the numbers in the table also give us a clue that the domain of educational big data keeps the vigorous growth.

**Table 4:** The keywords showed in the different periods.

**Timeline**	**Keywords**
2006–2011	Data mining, algorithm, computer, educational data mining, educational environment, curriculum, assessment, knowledge
2012–2016	Learning analytics, classroom, big data, blended learning, science, strategy, self-regulated learning, motivation, pedagogy, online, ethics
After 2016	Data analytics, challenge, college, integration, data privacy, analytics framework, course-design

### Countries/Regions Analysis

#### Co-authorship Analysis of Geography

**Figure 10:**
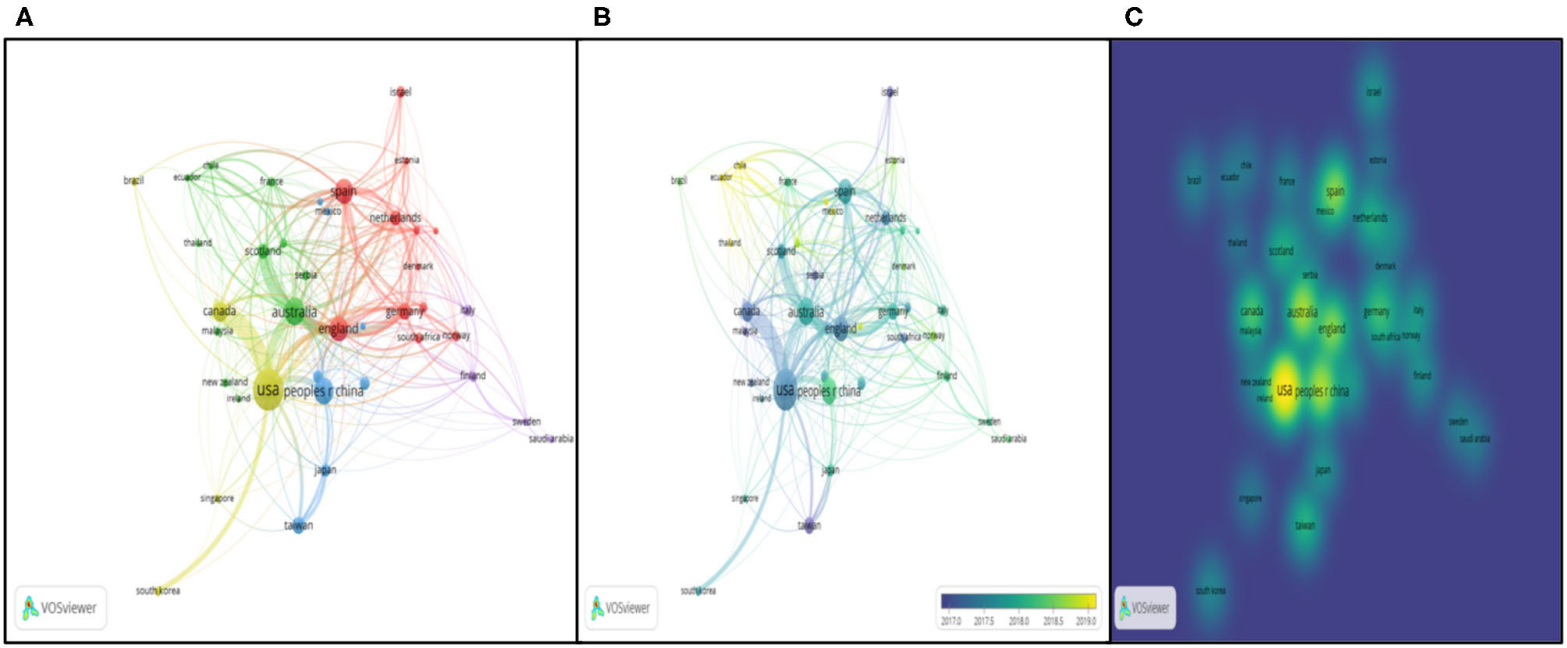
Co-authorship analysis of countries/regions. **(A)** Network visualization map based on document weights; **(B)** Overlay visualization based on document weights; **(C)** Density visualization based on document weights.

VOSviewer provides us with powerful functions to visualize the countries co-authorship in the bibliometric way. Setting the minimum number at 10, 40 countries of the total 88 met the threshold (as seen in Figure 10, links 302, total link strength 822).

In Figure 10A, the size of the nodes shows the number of documents, and different colors represent the distinct scientific camps. There are seven clusters totally. For instance, USA, Canada, Singapore, South Korea, Brazil and Iran are in the same cluster, which has the same research direction. Lines between two nodes indicate the link strength and cooperative relatedness. The link strength between the USA and China is 13, between the USA and Canada being 40, while link strength between England and Germany is 7. What can be indicated from results is that the implementation of cooperation can not only rely on geographical factors. Overlay visualization map is identical to Figure 10A, except the different colors with the color bar in the bottom right corner from blue to green to yellow. The bar shows document changes geographically during the different periods. From the map, Ecuador, Chile, and Thailand had latest contributions while highly productive countries like USA, Canada, Australia kept relatively low voice in the realm of educational big data. From Figure 10C, the density visualization network shows that USA, Austrian, English, China, Spain, Germany, and Netherland are the pioneers and leaders in cooperation in the domain of educational big data.

#### Citation Analysis of Geography

**Figure 11:**
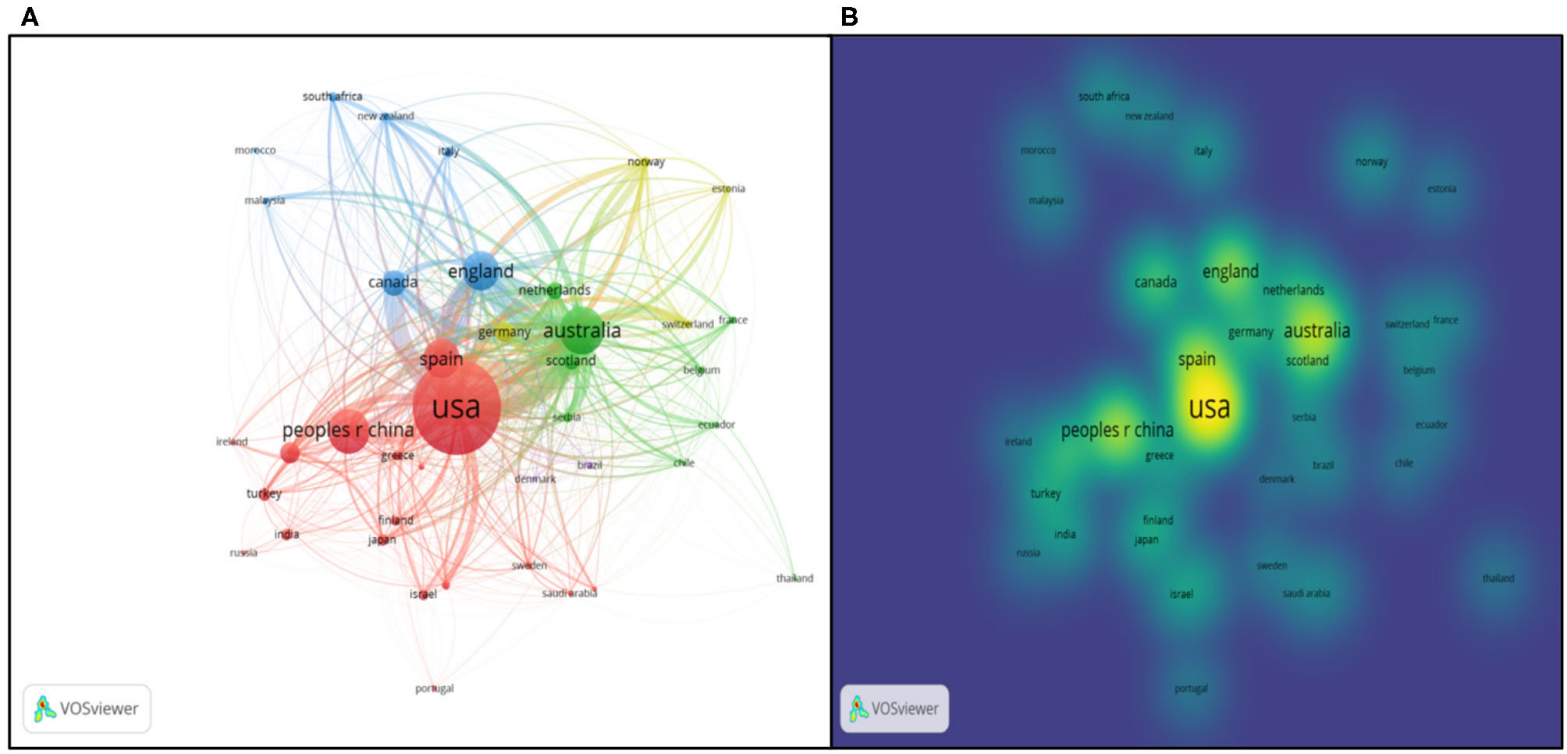
Citation analysis of geography. **(A)** Network visualization map based on citation weights; **(B)** Density Visualization based on citation weights.

Apart from above analysis in the co-authorship way, VOSviewer could also track the geographical data in the manner of citation. Co-citation refers to the relatedness of two contributors whose literature were simultaneously cited by another author (Zupic and Cater, 2015). Using VOSviewer, the authors set the threshold of 10 and got 40 countries of 88 in the co-citation visualization map (see Figure 11). The reference and journal co-citation analysis will be displayed in part institutions analysis.

Like above, the size of nodes represents the number of documents co-cited before and the distance between two nodes tells the scientific relations.

Australia, England, Canada, and USA kept strong cooperative links, while China, Turkey, Finland, and Japan had weak collaboration with others. It would be a wise idea for them to conduct more scientific research work with other countries in the future. The density visualization network concluded the main countries as USA, Australia, China, English, Canada, and Spain. Compared with Figure 10C, those countries with strong co-author relations generally hold large co-citation intensity. More specifically, the results of the geography analysis can be detailed in the Figure 12.

**Figure 12a:**
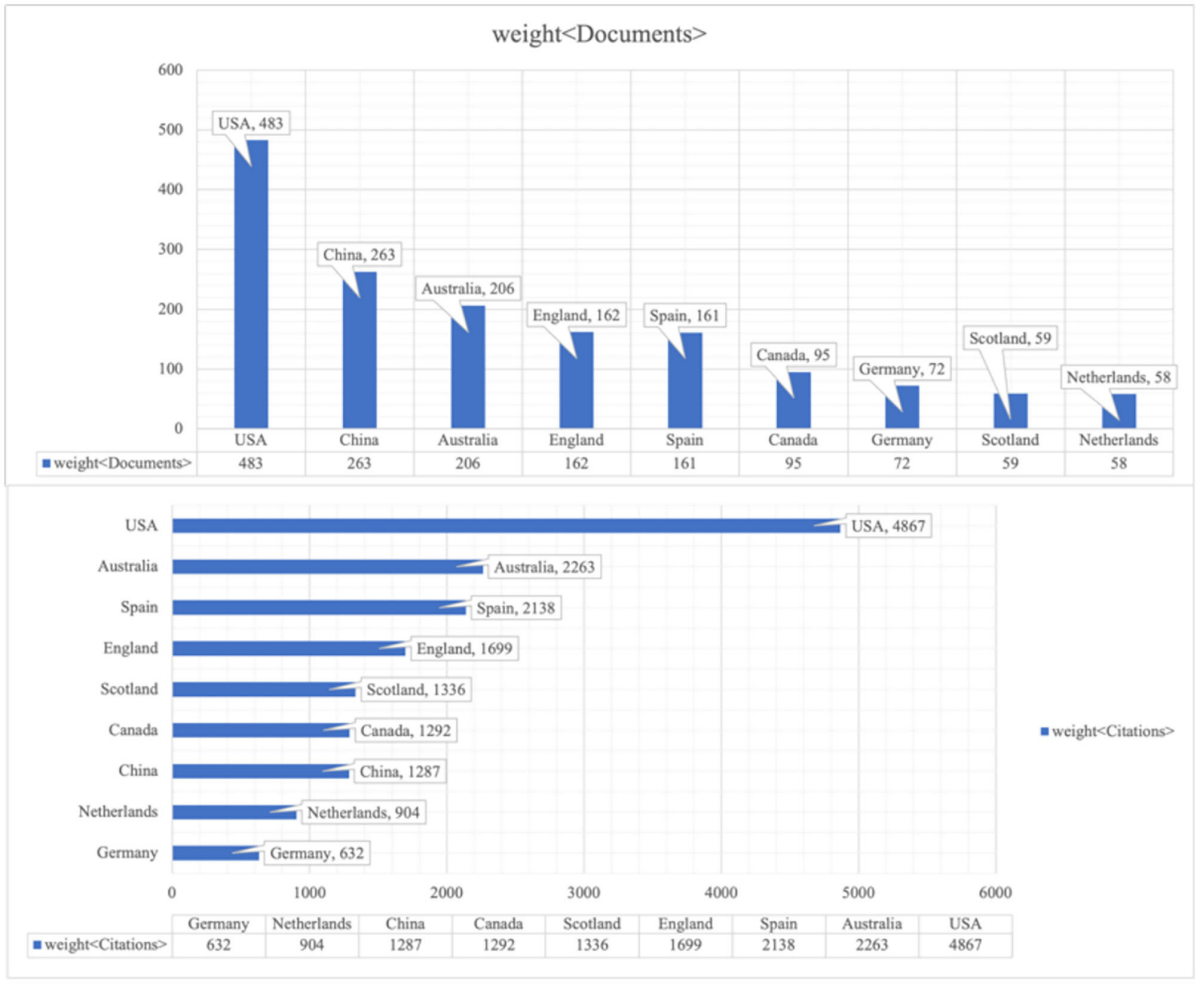
Weight of documents and citations of regions.

**Figure 12b:**
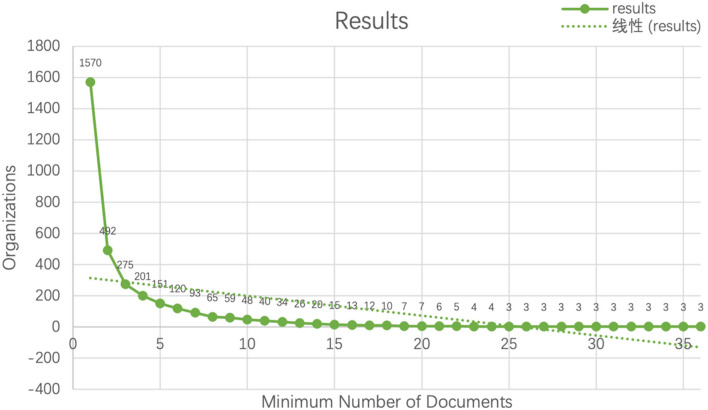
Range of different regions with different thresholds.

### Institutions Analysis

Before utilizing VOSviewer to map the institution network, different thresholds of minimum number of documents could generate distinct results (Figure 12). In the Figure 12, there was a sudden drop between 1 to 5 documents, which possibly indicated that the majority of scholars had only one or two publications and the study had already attracted great attention. In other words, for future successors in this domain, the investigation of big data in education still has a long way to go.

**Figure 13:**
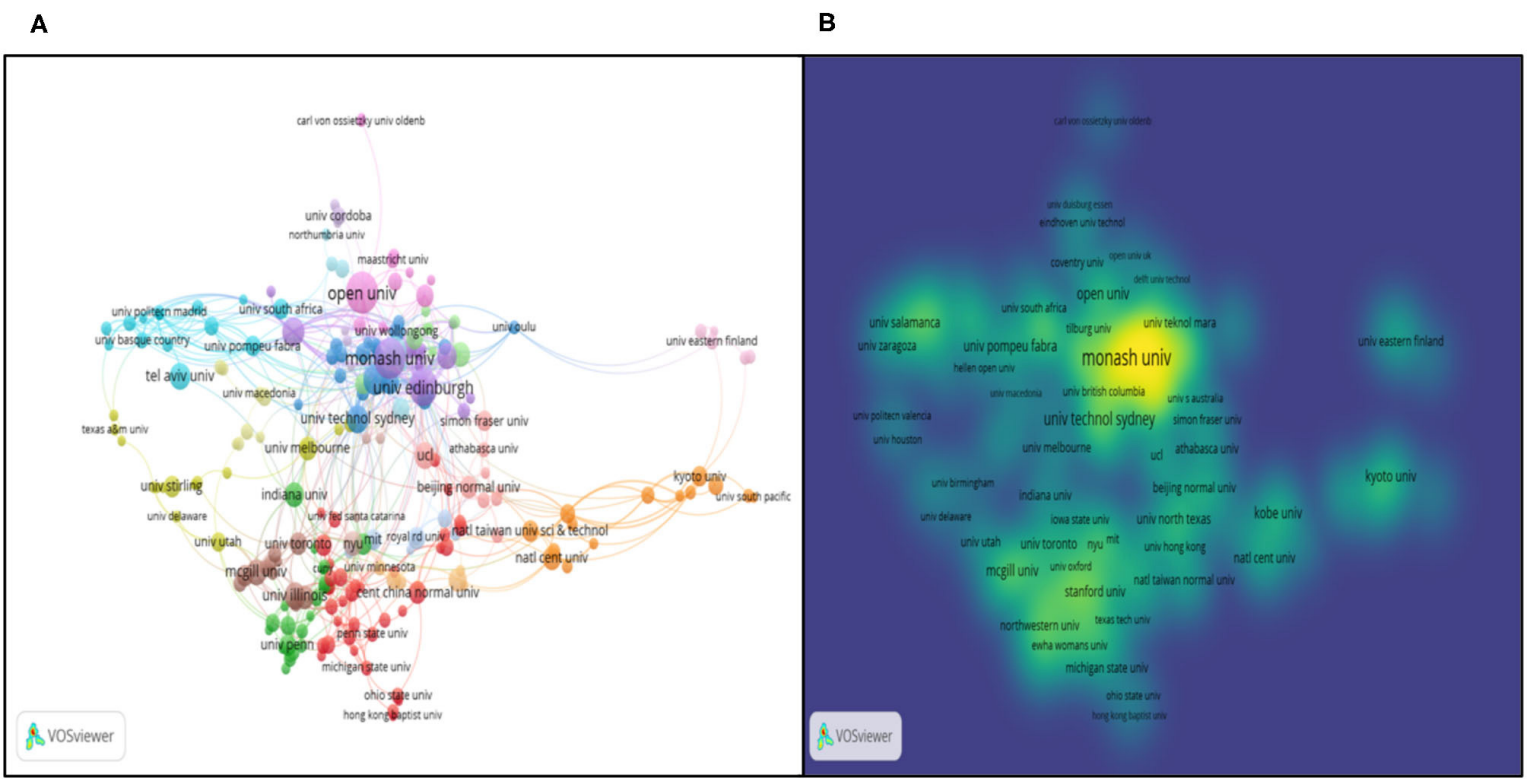
Co-authorship analysis of research organizations. **(A)** Network visualization map based on document weights; **(B)** Density visualization based on total link weights.

In Table 1, the top 10 organizations have been listed. In order to compensate the non-visual chart results, Figure 13 has presented the institution co-authorship network by VOSviewer both in network and density visualization way. From the network map, the authors made the conclusion that institutions generally have a high sense of cooperation on Education big data and keep tight academic relatedness. The Figure 13B shows the density map based on total link weights. From the trend of changing colors ranging from blue to green to yellow, collaboration of organizations in the European, Oceanian and North American region was much stronger than Asian countries, for instance Monash Univ (Australia, Oceania), Open Univ (England, Europe), Univ British Columbia (Canada, North America), Stanford Univ (UAS, North America) and Univ Edinburgh (Scotland, Europe).

### Co-citation Visualization Analysis of Reference

**Figure 14:**
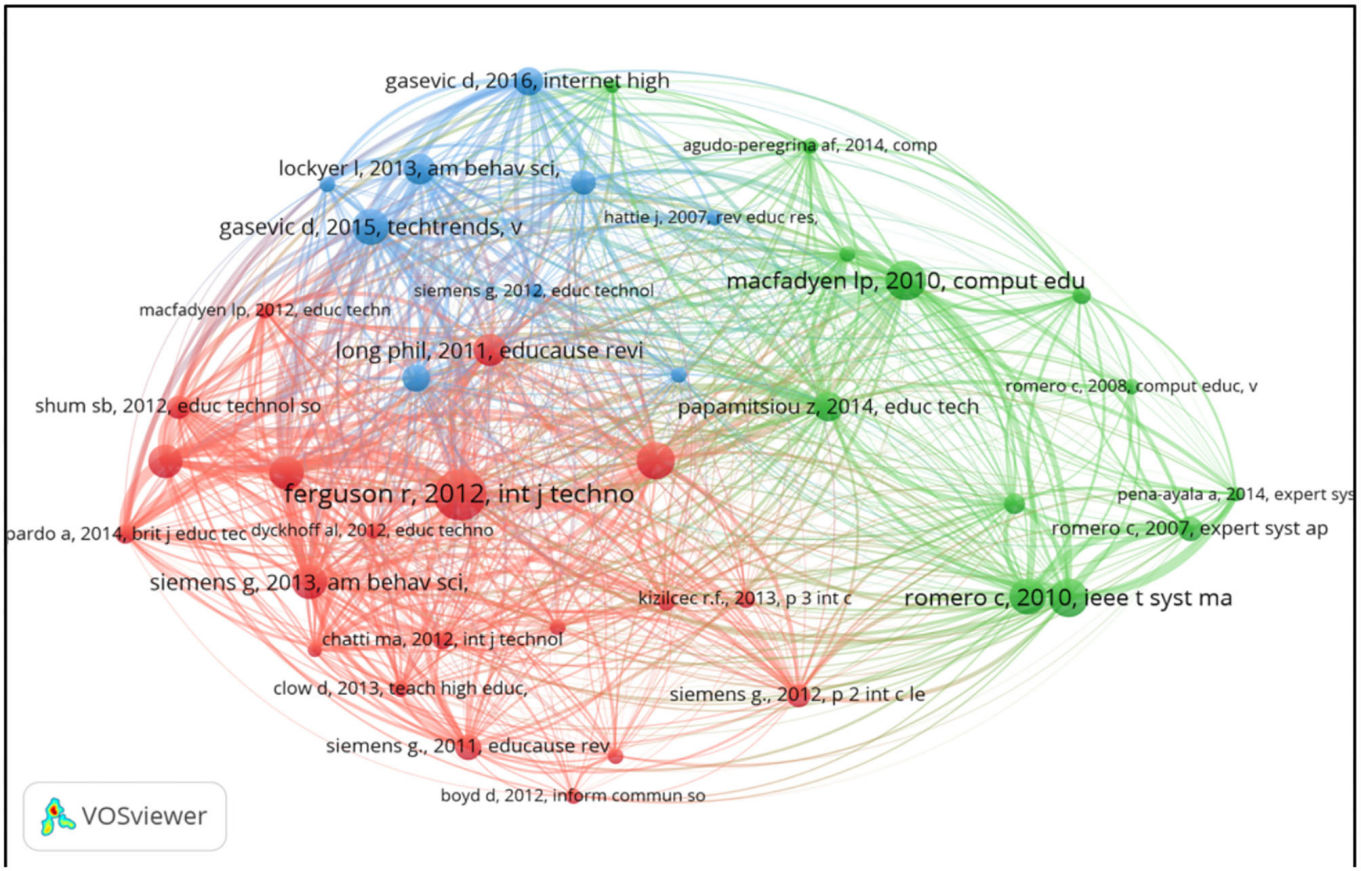
Network visualization map of cited reference [items 41, links 762, total link strength 4,936].

Co-citation is about co-cooperative relatedness when two papers were cited by the third paper (Boyack and Klavans, 2010), which is another index to survey the relevant literature in the bibliometric way. Unlike the method of citation analysis which focuses on the quality of subjects (including documents, sources, authors, organization, countries, etc.), co-citation could be used as a more scientific way to illustrate the collaborative pattern of research themes. Of the 55,947 cited references, the authors set the minimum number to get the results (Figure 14) and details the authors' information in Table 5. From the picture, the authors found that the biggest node was Ferguson (2012) about his theoretic contribution on learning analytics about drivers, developments and challenges published in *Int. J. Technology Enhanced Learning*, which stressed the relatedness of learning analytics, academic analysis, and educational data mining.

**Table 5:** The top 10 strongest co-authorship.

**Order**	**Label**	**Documents**	**Links**	**TLS**	**Citations**	**APY**
1	Gasevic, Dragan	35	37	108	1,107	2017
2	Rienties, Bart	24	11	35	252	2018
3	Pardo, Abelardo	21	19	59	502	2018
4	Dawson, Shane	14	13	39	763	2016
5	Williamson, Ben	14	2	2	361	2017
6	Munoz-Merino, Pedro J.	13	20	50	102	2018
7	Ifenthaler, Dirk	12	4	6	97	2017
8	Martinez-Maldonado, Roberto	12	16	25	84	2019
9	Knight, Simon	11	9	22	45	2019
10	Ogata, Hiroaki	11	10	22	60	2018

### Co-citation Visualization Analysis of Journal

**Figure 15:**
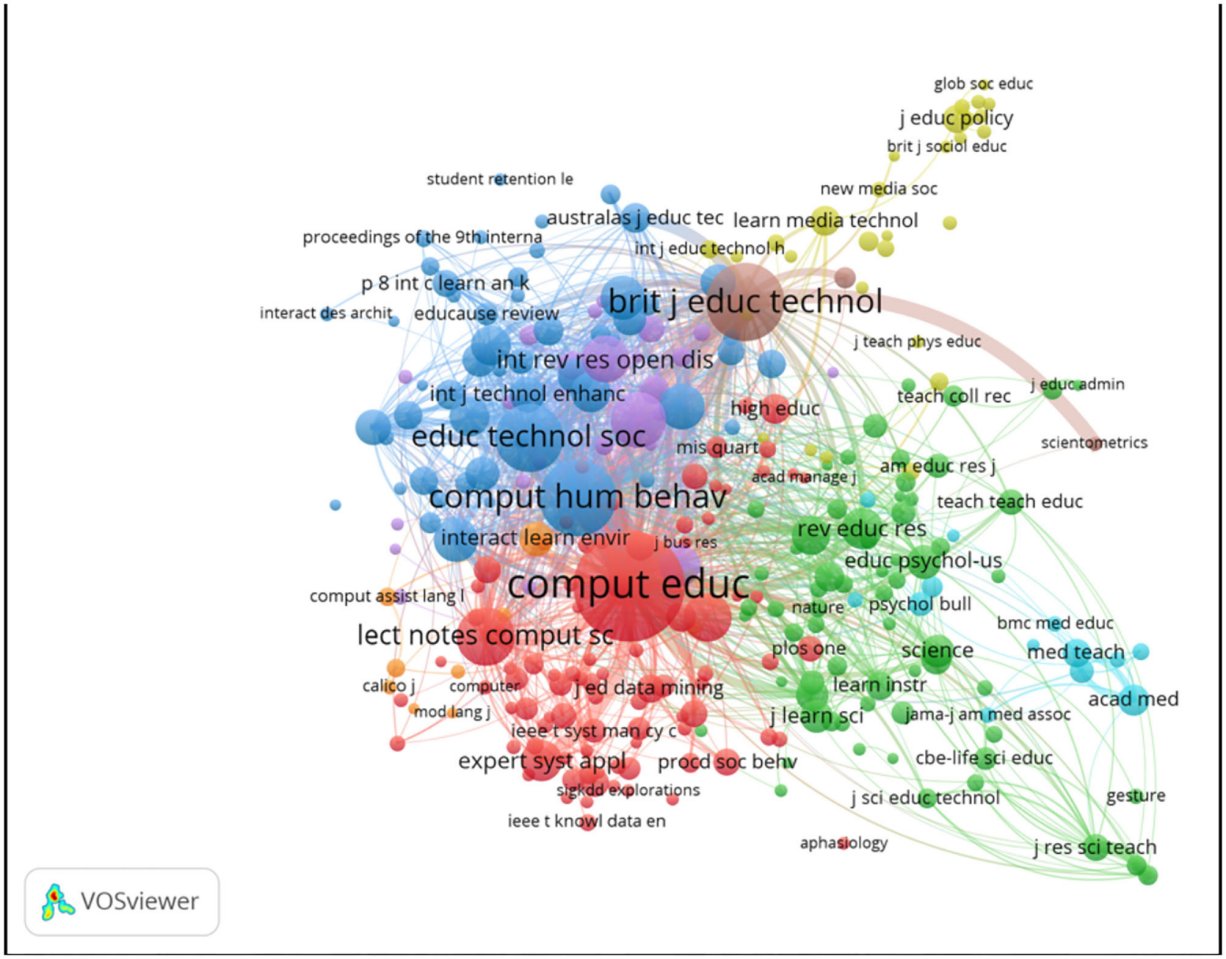
Journal co-citation network visualization map.

The authors set the minimum number of citations at 30 and visualized 289 journals of the 25,188 sources in Figure 15. The size of each node shows the number and contribution of that journal. The distance between two nodes also vividly represents the situation of link strength and citation. The distribution of each node also tells that different aspects in educational big data keep tight cooperation. In other words, successful implementation of big data in education cannot be separated from application of science technology and data analysis. In Figure 15, there are eight clusters, each representing distinct research subjects. “*Computers & Education,”* “*Lecture Notes in Computer Science,”* “*Expert Systems with Applications”* belong to cluster 1 indicating computer application. Cluster 2 has covered the research of educational psychology such as “*Journal of Educational Psychology,” “Educational Psychologist”* and “*Educational Psychology Review.” “Educational Technology & Society,” “ETR&D-Educational Technology Research and Development,” “American Behavioral Scientist” et al*. in cluster 3 underpin the importance of educational technology.

Table 6 lists top 10 most influential journals in the field of educational big data, *Computers & Education* enjoys the first priority in the order of citation. For instance, Kizilcec et al. (2017) once delivered a study of self-regulated learning strategies in *Computers & Education* to praise the digital learning environments for obtaining rich and large-scale educational data for scientific research and also pointed out several promising directions for data analysis in the domain of education, that is, the development of predictive models, feedback systems, and interventions with respect to Self-regulated learning strategies (SLR). In their words, anyone to address these challenges will put current research to a higher level and generate available strategies to underpin the platform of teacher/teaching.

**Table 6:** Top five most co-cited papers.

**Order**	**Author**	**Citations**	**Links**	**TLS**
1	Ferguson (2012)	134	54	605
2	Macfadyen (2017)	104	55	450
3	Romero et al. (2008)	101	52	342
4	Arnold (2012)	97	54	507
5	Gašević (2015)	96	53	490

## Conclusion and Implications

This research review adapted a bibliometric method to document and analyze the WOS database over the past 15 years. Utilizing science mapping analysis, the authors tracked the 1,708 articles from 2006 to 2021. This conclusion part provides interpretation of the review and offers implications for futural research.

### Interpretation of Results

The annual growth trajectory of publications unveiled that the number of papers fluctuated at low level during the initial periods between 2006 and 2014. However, after the critical period from 2012 to 2014, the trend showed exponential growth, probably because governments' initiatives to exploit the potential of big data, for example, the White House Big Data Report, Office of the Press Secretary in 2014 and the 2012 official report by the US Office of Educational Technology, Department of Education (Eynon, 2013). Apart from publication growth, the analysis of annual trend of citations also revealed the similar growth path. It is fair to state that big data in education receives and merits great attention from educational policymakers, administrators and educators.

The analysis of institutions exemplified that there was strong collaboration of organizations in the European, Oceanian and North American region, stronger than stronger than Asian countries. However, the results have also unveiled the fact that most research institutions lack outputs in quantity, giving the result of low thresholds of minimum number of documents. Enough scientific investigation on educational big data still needs to be on track.

The topographical analysis of research papers and institutions from WOS database uncovered the global geographic distribution with great contributions from UAS, Austrian, England, China, Spain, Germany and Netherland who also kept tight cooperation on the field. Optimistically, this regional imbalance has been redressed by the emerging countries from Asia (Thailand) and South America (Ecuador and Chile). Still, in some regions of Africa, due to economic situation and little opportunities of technology-assisted teaching or learning, there is little or no academic research on this realm, partly for lacking the international accessible knowledge about the most cutting-edge technology and application of educational learning analytics.

The co-citation analysis also identified the potential of integration of big data into educational practice. From the analysis of journal, computer and information science, technology analysis, ubiquitous network, and robust online education et al. All these foundations have transformed and improved how education itself functions. Educational settings have changed their ways to be more violent and drastic. In the era of big data, educators or educational administrators need to seize the initiatives to extract and analyze data for predicting and improving students' performance. This review also specified the core authors who have made groundbreaking and fundamental contribution on the field, for instance, Gasevic, Rienties, and Pardo.

Another contribution of this systematic review focused on distribution of keywords and timeline situation. From the vivid output, data mining, learning analytics, learning environment and psychology, the application of education, source of educational big data and users (or students) privacy were central to the educational big data research. To be more specific, the evolution also highlighted the shift from data mining to learning analytics to data analytics. Furthermore, apart from data-related analysis, researchers have also attached their attention from educational technology to individual educational psychology, more specifically, the psychological impacts on education, schoolteachers, students, school climate and even society. Interestingly, the application of educational technology inevitably raised the issue of ethics, particularly on privacy, which needs to be considered carefully and properly (Eynon, 2013).

To sum up, data mining, learning analytics, and algorithms highlight the shift to data analytics and educational psychology. Further, ethics, especially humanity privacy, provides new perspectives to rethink the potential of big data during educational activities and practices. Language program administrators and language teachers tether their efforts to the application of big data technology in the educational context, and psychological impacts should not be excluded when concerning the participants of school teaching or educating.

### Implications of the Results

The rise of modern technology and up-to-data social media contributes to information overload resulting in major stress in educational practice, which in turn probably causes serious psychological and mental disorders. It is high time to call for publica awareness of psychological problems and bridge this information gap in teachers. Several implications go after the findings from the visualization map of the objects.

#### Objectivity as Criterion in Data-Driven Educational Policy and Technology-Based Educational Growth

New data-based technologies tend to witness the era of objectivity in educational data application and scientific policy governance (Williamson and Piattoeva, 2019). Data play a central and vital role in the practices of educational policy implementing at local, national, and global scales. Several works have displayed the close interrelatedness between collection, circulation and analysis of educational digital data and dynamic sociotechnical networks of human, technologies, and policies providing new perspectives of evaluating education (Piattoeva, 2015; Hartong, 2016; Sellar, 2017). Meanwhile, psychological, and behavioral insights have been uttered in data-driven educational policy. Data cannot be treated as something entirely unified or sequential, instead we should consider as discourses and practices (Graham and Shelton, 2013). Big data has been subjected to the dilemma in which individual privacy and information circulation cannot achieve synergetic and simultaneous progress. Challenges of data privacy and ethics remain unsolved mysteries. The need to use the *politics of data* perspective to retreat education in the era of big data have also been stressed by some scholars (Halford et al., 2013). It is critical to make the social structure of big data visible, not as neutral fact.

#### New Perspectives to Regard and Practice Data in Educational Settings

Statistically, samples include certain huge amounts of population from which volumes of data can be gathered and received to assess the effectiveness of school reform and its associated effects on teaching development. Though the growing value of big data to education, many academic institutions keep slow pace with the implementation of big data projects (Macfadyen, 2017). Also, the collaboration between nations on educational big data has suffered from great geographic imbalance during the past 10 years. Hence, we assert the necessity for geographical diversity in the course of educational research. International educational administrators, educational philosophers, national policymakers, the school educators, educational institutions, and researchers, especially those inactive participants, need to address the importance of conceptualization of the possibility of introducing different technologies to extract and process information to underpin and improve the student learning. However, in its most negative forms, educational technology resulting in educational big data in school practice contributes to teacher stress and anxiety disorders, while in less aggressive forms, the application of technology can help adapt and innovate to the development-oriented conditions. Therefore, in step with the curriculum innovations and technology-preferred trend, those educational executors, especially school educators need to be more active to motivate their agency to unveil negative psychological conditions (anxiety, pressure, depression, fear, et al.) as explanation of skills lack, and positive psychological states (confidence, passion, pride, trust, etc.) as indexes of competence (Doménech-Betoret et al., 2017).

##### (1) Realize the Importance of Teaching-Learning Inter-Relatedness Between Learners and Teachers

School life and correlations with instructors significantly count in students' academic achievement. Valuing and promoting this close interpersonal relationship for instance, showing empathy and respect, becoming technologically available and psychologically allowed in discipline-coached proceedings, which also satisfied students' psychological need for achievement.

##### (2) Accomplish the Mission of Students' Inspection of Self-Ability

The big data area has challenged and positioned students subject to technical-assistance-inclination dilemma, which needs to re-perceive what skills they have gained from the former education and what achievement they have contributed after the evaluating feedback from guidance. This self-recognition of capacity also relates with students' psychological need for achievement.

#### Teachers' Role in Shaping Educational Development in the Big Data Era

After statistic survey, pedagogues can easily figure out the learning patterns and scientific rules about language learning which can be utilized to improve teaching pedagogy and effectiveness. Not only based on their teaching practice experiences, but educators can also utilize various educational tools (such as learning management systems, intelligent tutoring systems, e-books, MOOCs, etc.) in their teaching and educational contexts (such as practices of blended learning, flipped learning, or distance learning on math courses, language courses, programming courses, etc.) to meet the demand of professional development.

The COVID-19 outbreak that changed the traditional face-to-face way to impart school knowledge and up-to-data educational reform that altered the former educational practice in schooling put much more challenges and pressure on educators and administrators. Educators, especially school educational executors, are called to perform and activate their agency to improve their educational development in accord with the era of big data. The special year 2019 has greatly pushed teachers to know about and master the methods to introduce educational technology into daily teaching practices, which burdened themselves to some extent. To this end, teachers had to apply data mining technologies to extract information from participation in a discussion and use data analytics to examine students' learning state. All these unexpected outcomes would probably cause teacher anxiety disorders or stress, and in the end burnout in teaching, which calls more teachers to become more resilient people who can manage the negative factors to have positive consequences. Teachers are historically regarded as the effective role to shape students' experiences in school, also to constantly influence students' knowledge-acquisition skills and well-being. The ripple effects of teachers' assessment, interactions with students, and psychological impact on pupils' growth are necessarily targeted with difficulty, especially in the time of educational changes. The previous studies have focused on psychological interventions on students, but the students' passive environment testifies its insufficiency and effectiveness. Fortunately, teacher psychology serves more powerful and useful interests of educational development in the big data era.

**Figure 16:**
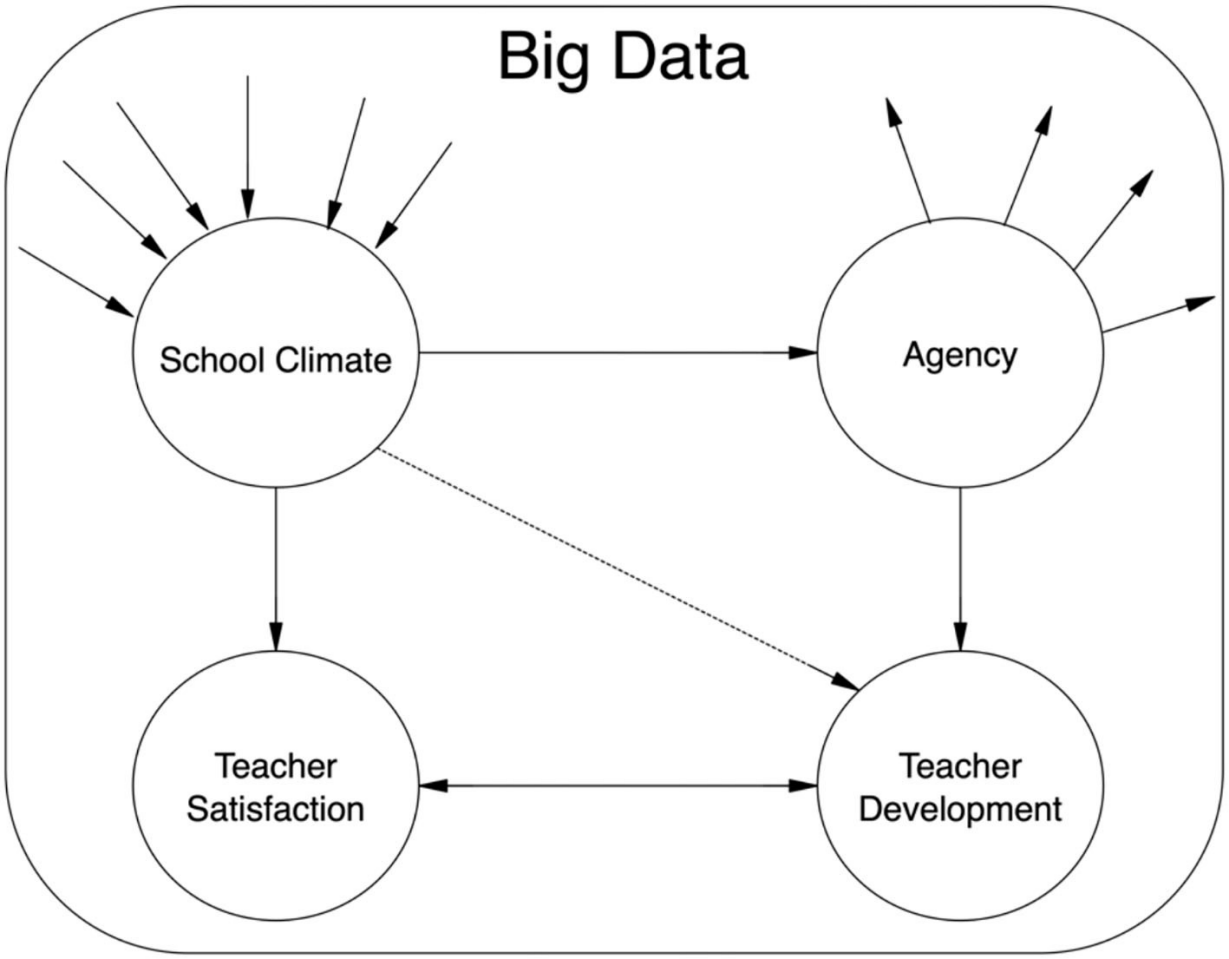
Relations of teacher development.

Teachers as active and dynamic participants, school educational environment and students play crucial and salient roles in the existence of school climate. Concerning the critical role of teacher in research, interactive ethnography paves the way for proceeding research design (Edwards, 2015), and normally discursive psychology, a model of research, is nominated as effective element in carrying out and performing agency in relation to dynamic school climate. Psychologically, teacher agency is a vital element to the successful implementation of educational innovation (Tao and Gao, 2017) in teaching practices. During educational development, educators are active, vigorous, and agentic contributors. However, the realization of positive educational growth is closely related to teacher agency. Consequently, compared with previous teaching, it might be more helpful for teachers to be given more opportunities to advance their personal agentic abilities. Hence, positive agency and teacher satisfaction resulting from pleasing school climate can lead to teacher development in the course of employment. What's more, the wide currency and growing-tendency of depression in education and living, lack necessary and productive cooperation in work, as well as the non-increasing rise in job satisfaction all suggest that the pressing need for synergy between positive emotion and education (school or lifelong). To utter it straighter and blunter, positive education, not only for knowledge-skill acquisition but also for achievement of sense of happiness, enables to increase resilience, positive engagement, and personal accomplishment, which also is highway to educational development (the relations shown in Figure 16).

Prior to elaborate the details in accordance with the variables in shaping teacher agency, many scholars have conducted scientific empirical studies to prove the pivotal role of agency within changes and constraints (e.g., Yang and Clarke, 2018; Yang and Markauskaite, 2021). Since the valuable contribution toward facilitating students' academic involvement and teachers' professional advancement, teacher agency has been shaped by the enables and constraints of educational changes under the impact of educational big data. In practice, most teachers would enact agency to reflect on and adopt preferred teaching modes to reveal the potent and innovative methods of blending traditional and incoming teaching approaches which often results in a bundle of challenges but also opportunities (Bryson and Andres, 2020). In the light of enactment, teachers' enactment of instruction, their knowledge and its guiding effect on teaching behaviors, their epistemology, and lastly their autonomy shape teacher agency to promote teaching quality and self-development which merit deeper explorations and investigations (Maclellan, 2017). Therefore, given teachers' professional agentic response and choices toward changes in the era of big data, it is expected for educational stakeholders to acknowledge the priority of teaches' digital competence and agentic participants in the teaching practice, also the need to discover the friendly and flexible interaction between agentic practices while teaching and the dynamic contextual resources (Gong et al., 2021).

#### Necessity of Bibliometric Approach

For those who have conducted the research review on this field adopting traditional methods of meta-analysis, we advise to add the complementary value by bibliometric approach. Science mapping allows us to extract the larger outcomes in the visual way among these piles of disordered and numerous literatures. Also, it has the ability to untangle some distinct features of contributors (authorship, co-citation, co-reference etc.). Therefore, we hold the idea of utilization of scientific bibliometric approach to showing research trends and foci vividly.

### Limitations

Although the scientific research method of science mapping complements the traditional bibliometrics models, it cannot completely take the place of these review methodologies which has provided great positive contributions on quality assessments. Our research strategy applied temporal analysis of some particular databases to unveil the temporal variations, which responds to the evolution of the field. Undoubtedly, it may drop some specific research questions unanswered in the course of study. Another limitation comes from the sources of data. We examined the database from Web of Science core collections and the articles are only focused on English. In other words, we have not covered the whole literature in the field.

## Data Availability Statement

The raw data supporting the conclusions of this article will be made available by the authors, without undue reservation.

## Author Contributions

All authors listed have made a substantial, direct and intellectual contribution to the work, and approved it for publication.

## Conflict of Interest

The authors declare that the research was conducted in the absence of any commercial or financial relationships that could be construed as a potential conflict of interest.

## Publisher's Note

All claims expressed in this article are solely those of the authors and do not necessarily represent those of their affiliated organizations, or those of the publisher, the editors and the reviewers. Any product that may be evaluated in this article, or claim that may be made by its manufacturer, is not guaranteed or endorsed by the publisher.
